# Intelligent Gas Sensors: From Mechanism to Applications

**DOI:** 10.3390/s25206321

**Published:** 2025-10-13

**Authors:** Jianghong Wei, Qing Peng, Yuee Xie, Yuanping Chen

**Affiliations:** 1 School of Physics and Electronic Engineering, Jiangsu University, Zhenjiang 212013, China; 2 Quantum Sensing and Agricultural Intelligence Detection Engineering Center of Jiangsu Province, Zhenjiang 212013, China; 3 School of Power and Mechanical Engineering, Wuhan University, Wuhan 430072, China; 4 IC Research Technology (Wuhan) Co., Ltd., Wuhan 430205, China

**Keywords:** intelligent gas sensors, IoT sensor networks, flexible sensors, machine learning, gas sensing technology

## Abstract

Intelligent gas sensors are indispensable devices widely used in modern society for environmental monitoring, healthcare, the food industry, and public safety. Recent advancements in wireless communication, cloud storage, computing technologies, and artificial intelligence algorithms have significantly enhanced the intelligence level and performance requirements of these sensors. Particularly in the Internet of Things (IoT) environment, flexible and wearable gas sensors are playing an increasingly important role due to their convenience and real-time monitoring capabilities. This review systematically summarizes the latest progress in intelligent gas sensors, covering conceptual frameworks, working principles, and applications across various fields, as well as the construction of IoT networks using sensor arrays. It provides a comprehensive assessment of recent advancements in intelligent gas sensing technologies, highlighting innovations in device architecture, functional mechanisms, and performance in diverse application environments. Special emphasis is placed on transformative developments in flexible and wearable sensor platforms and the enhanced intelligence achieved through the integration of advanced computational algorithms and machine learning techniques. Finally, a summary and future prospects are presented. Despite significant progress, intelligent gas sensors still face challenges related to sensing accuracy, stability, and cost in future applications.

## 1. Introduction

**Figure 1:**
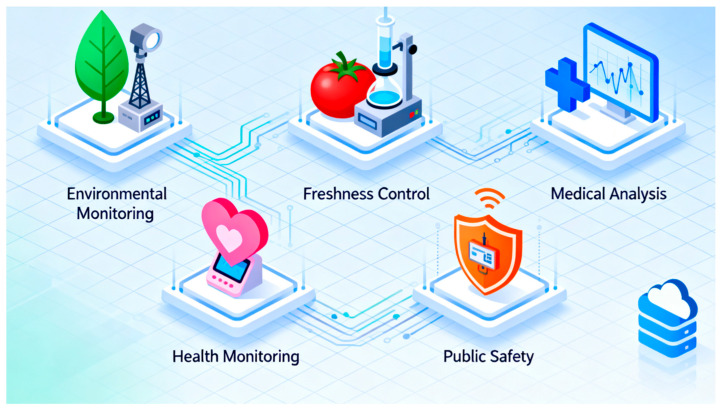
Smart gas sensors and their diverse applications in environmental monitoring, healthcare, industrial safety, and smart home automation.

A gas sensor refers to a sensing device capable of detecting changes in various components and concentrations in the environment [1]. By processing electrical signals generated from chemical or physical interactions between the target substance and the sensing material, these sensors provide critical information about the detected gases [2,3,4]. Over the past few decades, gas sensors have become an integral part of daily life [5], with applications ranging from atmospheric and indoor air quality monitoring to healthcare environment maintenance and pollutant tracking in the food industry, as illustrated in Figure 1 [6,7,8,9,10,11,12,13,14,15,16].

The rapid development of Internet of Things (IoT) technology and the growing demand for intelligent terminal devices have made intelligent gas sensors a focal point in numerous fields [17,18,19]. These sensors typically adopt a modular design and can be integrated into flexible wearable devices, portable instruments, and large-scale sensor arrays [20]. By combining communication technology with artificial intelligence (AI) algorithms, intelligent gas sensors enable more efficient real-time monitoring and analytical functions [21]. In the future, they are expected to play significant roles in digital home management, disease early warning systems, continuous non-invasive medical monitoring, and personal health tracking [22,23,24,25]. Additionally, they are increasingly used in food quality assessment, industrial leak detection, human–machine interfaces, and visual industrial safety monitoring [26,27,28,29,30,31,32]. Particularly promising are applications in plant gas analysis and live medicine, where they are poised to bring revolutionary changes [33,34,35].

Intelligent gas sensors are generally categorized into electrical and optical types. Electronic sensors have garnered widespread attention due to their compatibility with wireless communication modules and microprocessors, as well as their integration with standard electronic components [36,37]. These devices exhibit stable and sensitive performance, high portability, and support real-time monitoring and rapid analysis, making them valuable in both industrial processes and everyday life [38,39]. Electronic gas sensors convert gas information into readable electronic signals by leveraging the electrical properties of conductive materials such as semiconductors or multidimensional polymers [40,41,42]. Their operation relies on physicochemical interactions between target gas molecules and the surface of active sensing materials [43], ranging from weak reversible adsorption due to non-covalent interactions to covalent bonding. These interactions induce measurable changes in electrical properties (e.g., current or voltage) [44,45], which are processed and amplified by integrated electronic modules such as field-effect transistors (FETs) or metal-oxide-semiconductor (MOX) devices, enabling highly sensitive and selective gas detection [46,47,48].

Electronic gas sensors typically operate on principles such as changes in conductivity or resistance. Known for their high sensitivity and fast response times, they are ideal for portable devices [49,50,51]. The choice of conductive sensing material is crucial for sensor performance and reliability. Advances in flexible structural design and surface functionality have significantly improved sensor capabilities, allowing detection at parts-per-billion (ppb) concentration levels [52]. This advancement of nanotechnologies has made them increasingly suitable for various high-precision analytical and environmental monitoring platforms [53]. The separation of closely related species with minimal differences in physical properties but similar chemical signatures remains a key challenge to the successful operation of electronic gas sensors, especially within complex gas mixtures [54,55]. In order to improve sensor performance in such environments, material selection criteria need to be optimized strategically for the design, while signal processing algorithms developed may also help increase specificity and reliability in real-world application operating conditions [56,57].

Photoelectric sensors take a different non-contact optical signal readout approach to gas detection, focusing primarily on fluorescence and colorimetric modes of operation [58,59]. The sensing material interacts with the target gas of interest in a chemical manner, leading to detectable alterations in color or fluorescence intensity [60]. These sensors have a wide range of selectivity and give good discrimination between targeted gaseous species. Because its reliance on reactions of specific chemical substances (such as color changes or optical effects) makes it suitable for use in various environmental monitoring applications [61,62]. In general, photonic-based gas sensors exhibit high sensitivity, particularly for the detection of low-concentration gas mixtures [63,64]. Similarly, due to the complexity arising from uncertainty in identifying gas mixtures, accurate detection and quantification remain significant technical challenges for photonic sensor technology [65].

Intelligent gas sensor technology originated from William Levis’s micro-light irradiation detection method in the 16th century and underwent evolution from electrochemical-type sensors to digitalization and automation [66,67]. The MOX-SSA model introduced by the German company “SICONTROL” in the 1970s marked its widespread application in environmental monitoring [68]. With the advancement of computer technology and microcontrollers in the 1980s and 1990s, it achieved intelligentized management. By the early 21st century, the rise of IoT (Internet of Things) enabled sensors to integrate wireless communication capabilities, allowing real-time connection to cloud platforms and enhanced detection precision through artificial intelligence. Additionally, portable techniques and wearable platforms have provided efficient and universal solutions for precise chemical analysis [69,70,71]. These advancements address inherent challenges associated with human sensory limitations, labor-intensive sample per-treatment processes, and reliance on hazardous chemical reagents. Progress in the electronics industry has further accelerated the integration of miniaturized sensor chips with conventional electronic modules, driving the evolution of first-generation wearable devices [72,73]. This initial wave of wearable technology, often referred to as the wearable 1.0 era, was predominantly realized in rigid configurations, including smartphones, smart glasses, wristbands, and smartwatches. With the continuous growth of market demand for biometric information and wearable biometric diagnosis, along with the rapid progress of IoT technology, big data processing capabilities, and AI and robotics technology, the era of wearable devices is gradually shifting from the rigid wearable 1.0 form to the more flexible and intelligent next-generation wearable 2.0 era [74,75,76]. Future wearable devices will break through the limitations of current rigid chip and planar circuit platform technologies [77]. By adopting softening technologies such as textiles, patches, tattoos, and even mixed tissues, they will achieve a higher degree of skin adhesion, rollability, bendability, warpability, and stretchability, thereby significantly enhancing the applicability and user experience of the devices [78,79,80,81].

In the IoT ecosystem, this field mainly includes the following parts: The first component comprises flexible, skin-conformal wearable sensors designed for highly sensitive analyze detection and efficient signal transduction [82,83]. The second element is an integrated wireless communication module that processes and converts these signals before securely transmitting the data to cloud-based storage and computational platforms for advanced analysis and interpretation [84,85,86]. The third part is the AI training early warning system, which analyzes, interprets, predicts, and generates early warning information [87,88]. Meanwhile, non-wearable intelligent sensor arrays and gas sensors have been widely used in high-precision waste gas emission monitoring, detection, and early warning of dangerous and toxic gas leaks, mobile environmental monitoring, and law enforcement [89]. The smart wearable devices are gradually expanding into emerging fields, such as smart agriculture and non-invasive diagnosis, and through IoT technology, they show vigorous development potential in online medical care and early warning of epidemic events [90,91,92,93,94]. Flexible circuits are the core components of portable gas sensors.

The strategies for achieving stretchable and wearable functions mainly include three categories [95]. The first category is constructing flexible substrates by combining Au/Ag/Cu and conductive inks with rigid inorganic and soft organic semiconductor materials [96,97]. The second category involves directly bonding thin layers of conductive materials with low Young’s modulus onto flexible substrates [98]. The third category involves the preparation of solid stretchable conductors through methods such as mixing. Typical fabrication techniques involve processes such as photo lithographic patterning, screen and rotogravure printing, ink-jet deposition, and advanced additive manufacturing methods like three-dimensional (3D) printing [99,100,101,102,103]. The surface energy and roughness of different substrates, such as plastic polymers, cellulose paper, silk, or skin materials, vary greatly, directly affecting the gas sensors mechanical properties and adaptability [104,105,106]. For inorganic semiconductor materials, Metal oxides such as ZnO [107], SnO_2_ [108], WO_3_ [109], Sn-doped-Bi_2_O_2_CO_3_ [110], graphene [111,112], carbon nanotubes (CNTs) [113,114], disulfide transition metal compounds like MoS_2_ [115] and WS_2_ [116], MXene (e.g., Ti_3_C_2_Tx [117] and V_4_C_3_Tx [118]), phosphenes black phosphorus and purple phosphorus, there are various organic semiconductor materials including conductive ones conductive polymers metal–organic frameworks, Cu_3_(HITP)_2_ [119] and -Ni_3_ (HHTP)_2_ [120], covalent organic frameworks, hydrogen-bonded organic frameworks, HOF-FJU-1 [121], 8PN [122], and hydrogels [123,124] are widely used in the circuit and sensitive component design of gas sensors. These materials each have specific properties, such as Cu-Zn-Schiff-based polymers [125] or nickel-based hybrid materials [126], which can provide high sensitivity, reliability, and stability detection capabilities for gas sensors.

This review comprehensively discusses the operating principles, structures, transduction strategies, and detection capabilities of intelligent electronic and photonic gas sensors. Approaches to improving selectivity, accuracy, and sensitivity—including sensor array development, machine learning integration, and advanced functional materials—are covered. The review is structured as follows: Section 2 introduces the conceptual framework and working principles of gas sensors; Section 3 focuses on applications in environmental detection, healthcare, agriculture, food safety, public safety, and IoT; Section 4 provides a summary and future prospects. This work not only reviews state-of-the-art research but also envisions future directions and potential technological evolution in intelligent gas sensing.

## 2. The Conceptual Framework and Working Principle of Gas Sensors

### 2.1. Electronic Gas Sensor

**Figure 2:**
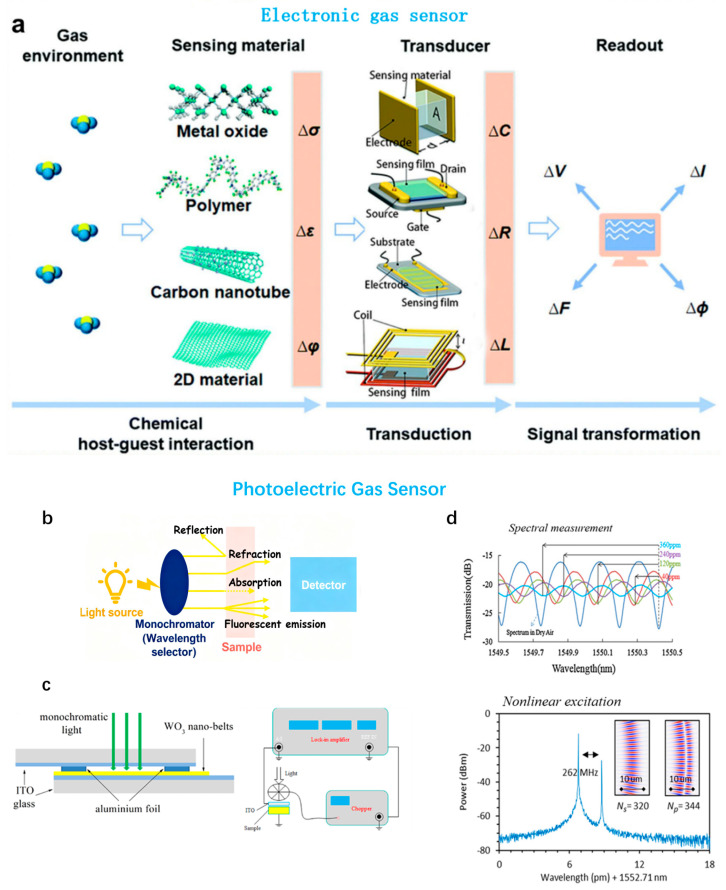
Illustrative schematics depicting the operating principles of electrochemical and optoelectronic gas sensors. (**a**) Structural layout of electronic gas sensors highlighting different transducer designs, including capacitive, field-effect transistor (FET), chemiresistive, and inductive configurations [134]. (**b**) Generic representation of a spectroscopic measurement arrangement [142]. (**c**) The structure diagram of photoelectric gas sensor [143]. (**d**) The gases and molecules adsorbed on the gas sensing platform are detected by photoelectric methods [144].

The electronic gas sensor primarily relies on semiconductor materials, such as WO_3_ and SnO_2_ [127,128]. When exposed to external gases, an oxidation–reduction reaction occurs at the surface of the sensor, leading to changes in electrical resistance that generate signals [129]. As an example, the specific working mechanism of a gas sensor based on WO_3_ is described as follows: when unknown gas molecules enter the detection area, they interact chemically with the semiconductor surface. Under oxidative conditions, tungsten in WO_3_ is oxidized to W^6+^. In anoxic environments, the target gas (e.g., hydrogen) can reduce WO_3_ to W^4+^ or other lower oxidation states. With the change in oxidation state, the electrical conductance of the sensor varies, resulting in current signals when an external voltage is applied [130,131]. The electronic gas sensor offers strong adaptability, functioning stably in high-temperature and high-humidity environments [132]. Its sensitivity is relatively reliable. However, it requires a continuous power supply and relies heavily on an external power source. This reliance increases the complexity and maintenance costs of the device [133]. An electronic gas sensor primarily consists of two fundamental elements: the active sensing medium and the detection unit (Figure 2a) [134,135]. The interaction between the sensing material and the target analyte in the environment mainly occurs through physical adsorption. Under the gas–solid interaction, its physical properties will change, including variations in electrical conductivity (Δσ), dielectric constant (Δε), and work function (Δφ), etc. The key components in electronic gas sensors, such as field-effect transistors, resistors, capacitors, and inductors, effectively convert these physical quantities into measurable voltage or current signals, which are usually presented in the form of current (ΔI) and voltage (ΔU) [136,137,138,139]. Table 1 summarizes the distinct properties of various transducer types employed in electronic gas sensors. In gas sensors, the physicochemical interaction between target gas molecules and sensing materials is the key mechanism for achieving high sensitivity and selectivity [140]. Specifically, this process mainly includes the following aspects: firstly, on the electron plane of the molecule, gas molecules transfer electrons with the carrier or active site on the sensing material, thereby triggering a local electrical signal; secondly, gas molecules may directly adsorb and undergo chemical reactions (such as REDOX reactions) on the surface of the sensing material, thereby altering the impedance characteristics of the sensor. For instance, in the interaction between nitrogen dioxide (SO_2_) and platinum-based sensors, SO_2_ acts as a reducing agent and reacts with Pb^2+^ on Pb(OH)_2_ to form PbS, thereby enhancing the conductivity of the sensing path [141]. For instance, in the interaction between methane (CH_4_) and Pt/GDC (palladium-diamond composite material), CH_4_ molecules combine with carbon components through the palladium surface, releasing electrons and generating intermediate oxides such as CH_2_O, which leads to local current changes. This process is often significantly influenced by factors such as temperature, humidity, and the surface activity of the sensing material, enabling gas sensors to accurately detect the concentration of specific gases. In addition, different types of gas molecules (such as CO and NO) may interact with sensing materials through different mechanisms, such as electron transfer or non-charge transfer methods [79,81].

Over the past few decades, the miniaturization and integration of rigid, solid-state silicon-based electronic sensors have driven a movement towards smart devices like smartphones, enabling a seamless user experience. This opened the door to new forms of flexible and wearable electronic sensors, leading to skin patches, electronic tattoos, or even luminous fabrics. These outstanding products have improved the comfort and convenience of interaction between people and computers. Although traditional rigid electronic sensors still have limitations in application, such as restrictions on size and application scope, flexible electronic sensors have opened up many new application scenarios thanks to their low-cost material production processes, such as printing technology and plasticity [145,146]. Against this backdrop, the collaborative advancement of sensing materials and flexible matrices has become a key factor driving the development of electronic gas sensors [147]. The base materials currently in use include common ones such as plastic foil, paper, textiles, and hydro-gels, but also cover a wide range of materials that can achieve self-shrinking, rollable, foldable, stretchable, and adapt to specific morphological changes [148,149,150,151,152]. Among them, a flexible sensor is a device that can withstand a bending curvature of up to 10^−1^ and a linear elastic deformation of more than 1%, and can maintain a normal working state without mechanical damage and significant performance decline. This definition provides clear guidance for designing and applying flexible electronic gas sensors. In terms of material selection, the ideal sensing material should possess three major characteristics [153,154]: first, it should have a high affinity for the target analyte to achieve significant binding ability and selectivity; second, it is a good surface exposure, providing sufficient conditions for the reaction; third, it is an appropriate electrical response characteristics such as significant changes in electrical conductivity, work function, or dielectric constant. The transducer part of the sensor includes components such as chemical resistors, FET, capacitors, and inductors, which convert the sensing signal into measurable and readable electrical forms such as voltage, current, dynamic, and frequency changes. As an important sensor component, the flexible substrate needs good mechanical toughness to meet the deformation requirements in various practical environments. This feature affects the sensor’s practical application effect and determines its service life and reliability in different scenarios. Among these, PVC and PET materials excel in lightweight, durable nature, cost-effectiveness, and flexibility, making them easy to process with lower production costs and suitable for long-term use. However, their temperature sensitivity is high; upon heat treatment, they cannot maintain their structure and performance simultaneously. Additionally, their chemical stability is not adequate, as certain gases (such as acidic or organic compounds) may react with plastics, diminishing the sensor’s reliability. These factors restrict the application of plastic-based materials in some highly demanding environments despite their cost-effectiveness. However, due to their cost benefits, they remain a preferred choice for foundational material selection. Fabrics offer a large surface area, facilitating the diffusion and absorption of gas-sensing reactions. They also possess excellent flexibility, suitability for attachment to complex surfaces, and superior durability for long-term use. However, they lack chemical stability and have poor conducting ability. Compared to the aforementioned materials, hydrogels exhibit excellent chemical stability and can maintain their structure in moist environments while preventing material exchange with the outside. They also offer shielding effects against many hazardous gases, making them ideal for protective or monitoring applications as a leading material for flexible gas sensor development. However, hydrogels are prone to deformation under external pressure due to their inadequate mechanical strength and thus require additional reinforcement through other means [155,156]. Through continuous breakthroughs in materials and technologies, electronic gas sensors are developing towards greater intelligence, convenience, and multi-functionality, providing rich possibilities for modern technological innovation.

### 2.2. Photoelectric Gas Sensor

Photoelectric gas sensors are typically based on optical principles, including absorption, scattering, diffraction, reflection, refraction, and luminescence, such as photoelectric, chemical, electro-chemical, and bioluminescence (Figure 2b) [142]. The working principle generally involves target gas molecules interacting with photons of specific wavelengths through absorption or scattering, altering their reflectance, or undergoing chemical reactions that trigger fluorescence or fluorescence quenching, thereby generating detectable signals [157]. The photoelectric gas sensor offers high sensitivity, particularly providing significant color changes in dry environments. However, its performance diminishes significantly under high-temperature or high-humidity conditions, and it demonstrates limited environmental adaptability [158]. Among them, colorimetric and fluorescence sensors have been widely reported in gas sensing due to their utilization of the characteristics of intermolecular interactions [159]. Photoelectric gas sensors based on chemically reactive colorants do not rely on the physical properties of the target analyte but capture the chemical sensing signals between it and the chromophores or fluorophores, which enables them to distinguish between very similar analytes effectively. This method overcomes the limitations of traditional methods based on physical adsorption or non-specific chemical interactions. At the same time, it utilizes the photoelectric selection and discrimination capabilities of intermolecular interactions ranging from weak van der Waals forces to strong covalent or ionic bonds, significantly enhancing the detection sensitivity and specificity [142]. Figure 2c presents the structural schematic of the photoelectric gas sensor.

The analytical technology used to design photoelectric gas sensors brings together optical and electronic characteristics for effective identification of different gases. It usually comprises four key components: a visible or ultraviolet light source, a wavelength-selection unit, a functionality sensing substrate, and a wavelength-sensitive detector directed at the target analyte [160,161]. By combining these elements in cases that use array-based configurations with high-end digital imaging methods, the sensor can perform accurate detection and classification of sophisticated odorous profiles. These generated color difference patterns represent unique visual fingerprints of individual odorants in a manner analogous to olfactory specificity, where enhanced specificity is achieved by response pattern analysis [162,163]. In this respect, the photoelectric gas sensors have several clear advantages in many possible use cases (Figure 2d). Current research efforts are being dedicated to producing photoconductive elements that can be deposited on a range of substrate materials to satisfy performance requirements in different applications. A rather unique feature about this set of photoactive sensing materials is its successful use in multiple platforms, such as paper-based substrates, polymeric films, hydrogels, and silicone matrices, which make them a suitable candidate for flexible electronics and wearable technologies [164,165,166,167,168]. This technology provides photoelectric gas sensors with additional practical flexibility and adaptability.

The important point behind their operations is the color model that provides a mathematical model for representing and standardizing the color properties. They are basic models for color variation comprehension, visualization, and analysis, which are crucial to many optical and electronic systems, particularly in colorimetric gas sensing [169]. Commonly used color models are International Commission on Illumination (CIELAB), Red, Green, Blue (RGB), Hue Saturation Value (HSV), Cyan Magenta Yellow Black (CMYK), and YIQ and YUV. CIELAB and RGB are the most common color spaces used in colorimetric gas sensors because of their ability to convert color changes into quantitative signals. Color transformations in these models are explicitly parametric, effectively encoding a perfect analytical relationship of analyte concentration to color change. Colorimetric gas sensors determine variations in light intensity across several wavelengths and then convert them into a color response through some calculations to quantify target analyte levels. The color model-based approach widely employed in industrial and medical sectors enables the conversion of complicated optical phenomena into digital signals, which can be easily read and processed to analyze accurately.

The color model is a mathematical framework for describing and analyzing colors and plays an important role in applying optical technology. Based on the intensity changes in the three colors RGB, the RGB color model is widely used in color perception measurement due to its intuitive 3D spatial representation. However, since the range of each channel is relatively single, 0–255, some color information may be omitted or distorted [170]. Therefore, methods and techniques combine multiple channels to enhance resolution and accuracy, such as calculating Euclidean distances, ratio analysis, and more complex combined models. In addition to RGB, the CIELAB color model is based on luminance (L) and two dominant color channels, A (red/green) and B (blue/red), which can more intuitively reflect the color perception in human vision. The advantage of the CILAB model is that it is consistent with various devices and environments, and using Euclidean distance (ΔE) to calculate the color difference can better describe the change in color space. As for the HSV color model, dividing by hue results in a more consistent performance across different lighting conditions.

### 2.3. Gas-Sensing Platforms Material

Gas-sensitive materials are crucial as the base material for gas sensor fabrication in determining a gas sensor’s performance. Ideally, the sensing materials should have a large surface area to enhance effective and selective absorption of gas molecules while converting gas information into detectable signals, along with excellent mechanical performance. Over the past few decades, numerous gas-sensitive materials have been developed, such as carbon nanotubes (CNTs), silicon carbide (SiC), metal oxides, graphene (Gr), metal polymers, and metal–organic frameworks (MOFs). Their exceptional properties have opened up vast prospects for their application in the gas sensor field.

Carbon nanotubes (CNTs) possess tubular structures and can be visualized by rolling graphene (Gr) sheets into cylindrical forms [171]. Since their discovery by Ijima and colleagues, these materials have gained widespread application across various fields due to their unique shape and excellent mechanical, electrical, chemical, and thermal properties [172]. CNTs are highly favored in the gas-sensitive field owing to their chemical stability, high surface area-to-volume ratio, and inherent large surface-to-volume ratio. However, their selectivity for specific gases remains inadequate [173]. A key strategy for enhancing selectivity and sensitivity is functionalization. The integration of a 2D SWCNT network onto a 3D structure has been considered an effective approach to increase the surface density of SWCNTs and improve their gas-sensitive properties. Li et al. reported a field-effect transistor (FET)-based gas sensor constructed from F8T2 and highly purified semiconducting SWCNTs, capable of detecting NO_2_. Through the formation of 3D SWCNT networks in F8T2 matrices, the absorption, aggregation, and retention properties of NO_2_ were significantly enhanced [173]. This resulted in the development of an amino-functionalized carbon nanotube sensor for detecting three gases (CO_2_, SO_2_, and NO_2_).

Metal oxides possess adjustable active sites on their surfaces, providing efficient adsorption and reaction platforms for specific gases and optimizing detection performance [174]. Additionally, metal oxides are capable of accelerating the oxidation or reduction reactions of gases, thereby enhancing the ability of gas sensors to respond quickly to gas concentrations. Furthermore, metal oxides exhibit excellent compatibility with other materials, enabling the formation of high-performance sensor components along with various conductive materials for the design of multifunctional gas detection systems [174,175]. Moreover, metal oxides are characterized by thermal and chemical stability, nontoxicity, biocompatibility, a strong affinity for toxic substances, and a relatively high carrier mobility rate [176]. These properties make them favorable candidates for the preparation of gas-sensitive platforms. In general, gas sensors based on metal oxides are known for their chemical inertness and resistance to harsh environments, where the material’s electrical properties, such as its resistivity, change upon exposure to target gases [177]. The primary factor influencing resistivity changes is either the availability of surface oxygen ions or the volume of the material itself. Gas sensors detect the sensing mechanisms for oxidizing and reducing gases through interactions between conductive electrons and these target gas molecules, leading to changes in the electrical properties of the active material [178,179]. The temperature (T) significantly influences the effectiveness of oxygen ion species chemically adsorbed on the surface of metal oxide materials, as illustrated by Equations (1)–(4) [180],

(1) $$O_{2}(gas)\to O_{2}(ads),$$

(2) $$O_{2}(\mathrm{ads})+e^{-}↔O_{2}^{-}(ads);T<100\;{}^{\circ}C$$

(3) $$O_{2}(\mathrm{ads})+2e^{-}↔2O_{2}^{-}(ads);T\sim (100\;{}^{\circ}C\sim 300\;{}^{\circ}C)$$

(4) $$O^{-}(\mathrm{ads})+e^{-}↔O_{2}^{-}(ads);T>300\;{}^{\circ}C$$

When energy is provided through heat or light, incoming gas molecules can effectively overcome activation barriers and interact with the source-sensitive materials, thereby enhancing sensing performance. To improve the sensing performance of metal oxide-based gas sensors, a significant research trend has been to convert thin-film sensors into sensors with layered nanostucture (NS) [181,182]. Additionally, the enhancement of sensor performance also depends on certain fundamental material properties, such as bond types (e.g., covalent bonds), active sites, and surface atomic arrangements. The key factor in determining these properties lies in the formation of crystals with different energy levels on the material’s surface.

Resistive gas sensors operate based on the change in resistance when exposed to various gases. Different types of materials, such as graphene, carbon nanotubes, metal–organic frameworks (MOFs), and conductive polymers, have been utilized for resistive gas sensor implementations [183]. Gas sensors based on semiconducting metal oxides are preferred due to their advantages, including high sensitivity, high stability, short response/recovery times, simple operation, compact size, low cost, etc. However, they suffer from poor selectivity and typically require high temperatures to achieve optimal sensing performance [184]. To address these issues, noble metal functionalization has become a popular solution. Various noble metals, such as Pt, Pd, Au, and Ag, have been widely used for modifying MOS structures, with different techniques, including chemical reduction, electrochemical reduction, e spin-on, UV illumination, and spray method, employed to disperse noble metal aggregates on the sensor layer surface [185,186]. The enhancement in gas response by these metals can be attributed to both electrons and CS effects. In the first case, upon exposure to air, some metals form metal oxides that act as strong electron acceptors. Consequently, an intense electron depletion layer is induced near the sensing material interface. When exposed to reducing gases, the oxides are reduced into metals, which then transfer electrons back to the sensing layer, thereby relaxing the electron-depletion layer and significantly enhancing gas response. The oxide-to-metal transition in this process generates sensor signals. On the other hand, CS is related to the catalytic activity of metals [187]. Indeed, oxygen molecules can easily decompose on metal agglomerate surfaces and transfer via a so-called overflow effect to adjacent metal oxides, leading to additional oxygen absorption at the sensing layer surface within shorter time spans. Hydrogen molecules also tend to dissociate and migrate to the oxide surface from metal surfaces. Both mechanisms can enhance sensing by enabling more oxygen adsorption on the sensor layer surface in shorter times. Additionally, since the work function of noble metals is generally higher than that of the sensing layer, a Schottky junction forms at their interface when the sensor is exposed to target gas environments. The interaction between the gas and the oxygen ions adsorbed on the sensor surface triggers electron release back to the sensor surface, causing a significant reduction in the Schottky barrier height, thereby enabling resistance adjustment of the sensor.

## 3. The Application of Intelligent Gas Sensors

Intelligent electronic photoelectric gas sensor technology has developed rapidly in recent years. Its inherent attributes of superior detection efficiency, operational simplicity, and ease of system integration have facilitated extensive deployment across diverse sectors. These include monitoring atmospheric pollutants for environmental protection, diagnosing medical conditions through biomarker detection, assessing food quality and spoilage, and providing early-warning systems to enhance public safety. This type of sensor can monitor the concentration changes in target gases or substances in real time at low cost, high sensitivity, and fast response rate, and has shown significant advantages in areas such as environmental pollution source tracking, early disease screening, food quality control, and abnormal behavior identification during travel.

### 3.1. Environmental Pollutant Detection and Monitoring Strategies

**Figure 3:**
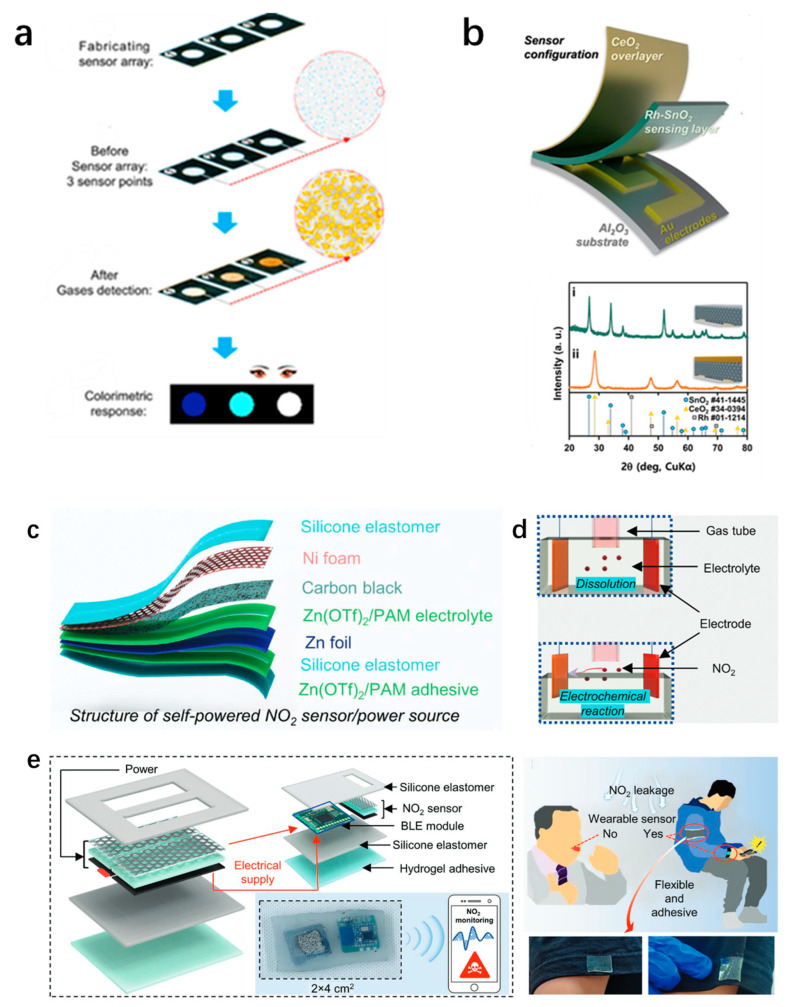
Flexible and wearable electronic gas sensors based on metal/metal oxide and hydrogel platforms for environmental pollutant monitoring. (**a**) Colorimetric visualization of aldehyde gases achieved through a silver-functionalized paper sensor array [189], (**b**) ultrasensitive differentiation between VAHs and non-VAHs utilizing a CeO_2_/Rh–SnO_2_ bilayer sensor [190], (**c**–**e**) wireless, self-powered NO_2_ gas sensor integrated into a flexible hydrogel patch for real-time environmental monitoring [16].

Formaldehyde (HCHO) is one of the most concerning chemical substances among indoor environmental pollutants, and its potential threat to human health has attracted widespread attention worldwide [188]. The World Health Organization (WHO) reports that chronic exposure to environmental formaldehyde concentrations exceeding 0.08 ppm is associated with an elevated risk of carcinogenesis. Consequently, accurate and rapid detection of formaldehyde is essential for environmental safety and public health protection. Portable electronic gas sensors, particularly those utilizing metal and metal-oxide-based catalytic materials, have emerged as preferred analytical tools owing to their remarkable sensitivity, high selectivity, and capability for real-time on-site monitoring. However, such sensors still face many challenges in practical work, including the inability to achieve international standard detection accuracy and carbon monoxide poisoning caused by waste gas emissions during long-term use, significantly limiting their practicality in complex environments. Yang et al. developed a paper-based colorimetric sensor array functionalized with silver nanoparticles (Figure 3a) [189]. This innovative platform enables rapid visual detection and effective discrimination of five commonly encountered trace aldehyde gases at the molecular level. Through an efficient reduction reaction, silver ions are reduced to nano-silver. This colorimetric sensor array provides a new method for continuous, ultra-sensitive, and visual detection of trace air pollutants. The detection limits (LOD) of the five aldehyde gases are as follows: Formaldehyde (FA) 9.0 ppb, acetaldehyde (AA) 3.1 ppm, propionaldehyde (PA) 3.5 ppm, glutaraldehyde (GD) 23.8 ppb, hydroxyformaldehyde (HF) 71.5 ppb. Importantly, the LOD values of these thresholds are significantly lower as compared to their corresponding PELs, demonstrating the high sensitivity of our sensing platform. In addition, each aldehyde also gave a characteristic visual fingerprint colorimetric response profile, which ensured accurate and facile discrimination of analytes.

VAHs belong to the class of trace air pollutants that are widely recognized as highly toxic and causing toxicity effects on both human health and the environment. Due to their ubiquity and adverse health outcomes in indoor environments, it is crucial to have particular methods capable of efficiently detecting these compounds. On the other hand, metal oxide semiconductor (MOS)-based chemiresistive sensors are most popular in this regard as they operate at high temperatures, which increases the sensitivity of adsorbed oxygen species on the sensor surface towards VAHs. This interaction promotes redox charge transfer processes, which result in an electrical resistance change that can be measured for detection purposes [190]. Nevertheless, these sensors remain inherently flawed with poor selectivity and may be subject to cross-contamination by other reactive gases such as formaldehyde and ethanol. However, the nature of these tasks constrains utility in complex and dynamic environmental settings. Lee et al. fabricated a dual-layer chemiresistive sensor array based on CeO_2_ and Rh–SnO_2_ that is capable of differentiating aromatic gases in the presence of non-aromatic interferents (Figure 3b), as well as allowing classification while offering semi-quantitative detection of VAHs [190]. The experimental results confirmed a significant increase in sensitivity toward VAHs with the sensor array as compared to pristine SnO_2_, which was mainly attributed to the mild catalytic effect of CeO_2_ overlayer that kinetically expedited surface-limited reaction and resulted in enhanced differentiation of signatures. This dual-layer configuration promotes the conversion of highly reactive interferent gases, e.g., ethylene and methanol, to less reactive or inert species, which prevents interference with the target analytes. This architectural design not only elevates the detection sensitivity to aromatic gases but also effectively ameliorates their responses to background environmental pollutants. As a result, the sensor exhibits excellent robustness and selectivity, making it a desirable prospect for reliable air quality monitoring.

Nitrogen dioxide (NO_2_) is a prevalent inorganic gaseous pollutant that contributes significantly to the formation of acid rain and photochemical smog, posing serious environmental and health concerns. Numerous studies have explored advanced electronic sensing materials for trace NO_2_ detection, encompassing both rigid and flexible platforms, including transition metal dichalcogenides (TMDs) [191], MXenes [192], phosphorene [193], and metal–organic frameworks (MOFs) [194]. Despite these advancements, substantial technical challenges remain, hindering their widespread practical application. On the one hand, the recovery difficulties caused by the high physical adsorption energy and desorption problems of -NO_2_ chemical resistance and FET sensors, as well as the deterioration of materials at high humidity. On the other hand, the flexible nitrogen dioxide sensor has poor ductility under mechanical deformation, coupled with the flexible substrate. Wu et al. developed a flexible electrochemical sensor based on zinc trifluoromethanesulfonate [Zn(OTf)_2_]/poly(N-propyl acrylamide) (PAM)/C-MoS_5_ composites (Figure 3c–e) [16]. They further investigated hydrogel-based sensing platforms, which demonstrated exceptional performance characteristics, including ultra-high sensitivity (1.92%/ppb), an ultra-low detection limit of 0.1 ppb, high recovery efficiency, and reliable operation under mechanical deformation, sub-zero temperatures, and high-humidity conditions. These properties establish hydrogel-based sensors as promising candidates for NO_2_ detection. Moreover, the inherent flexibility of these sensors enables seamless integration into compact circuit modules, facilitating the development of wearable wireless NO_2_ monitoring systems for real-time environmental surveillance and early-warning applications.

### 3.2. Healthcare Application

**Figure 4:**
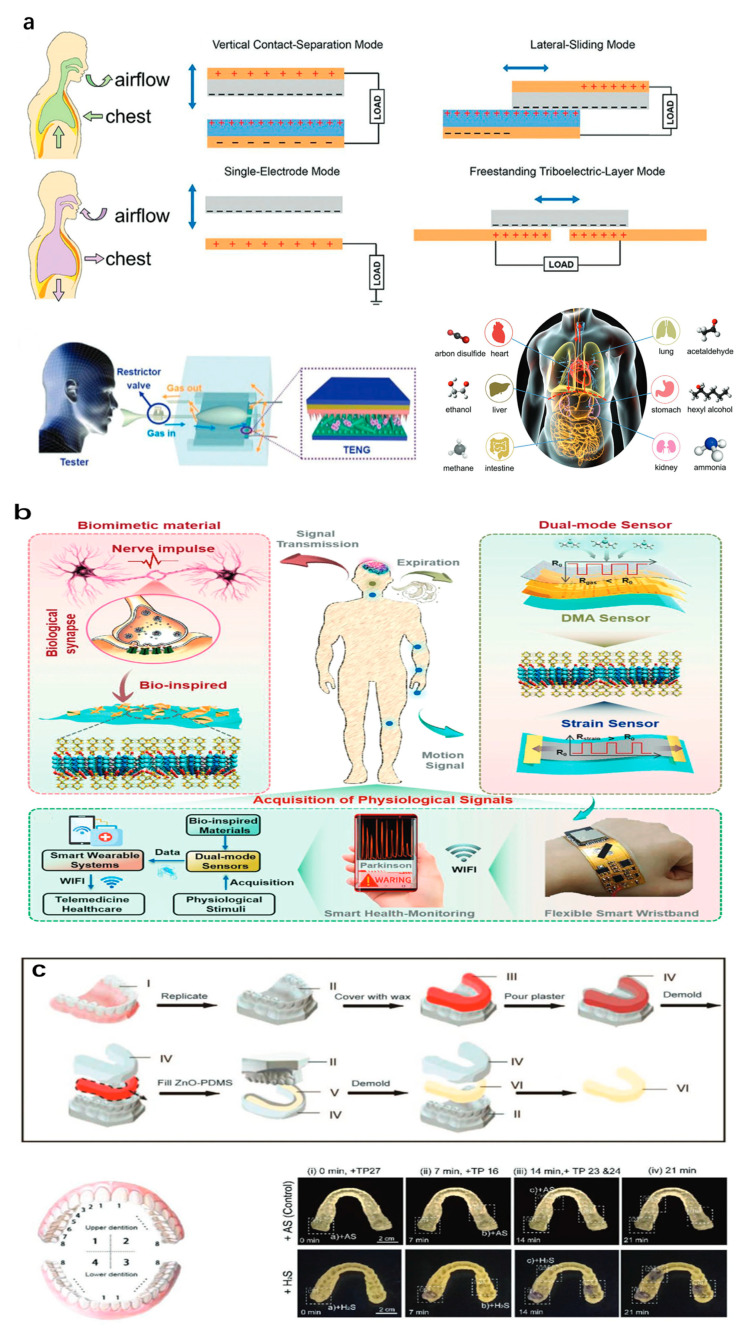

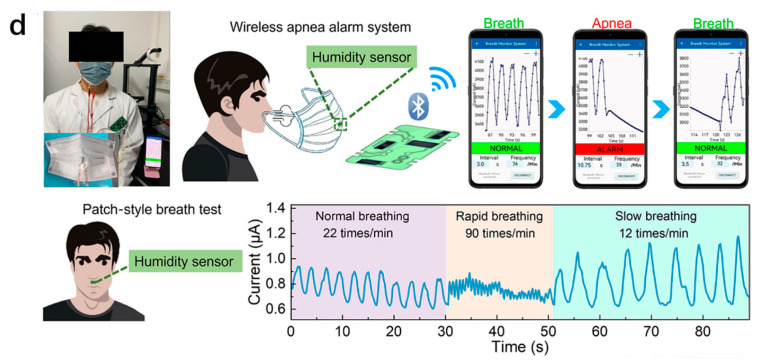
Advanced multifunctional gas sensors designed for the detection of disease-associated pathological biomarkers. (**a**) Examples of exhaled biomarkers used for clinical diagnostic evaluation [196]. (**b**) A flexible, dual-mode device integrating gas and strain sensing for point-of-care health monitoring in Parkinson’s disease patients [200]. (**c**) A wearable, fluorescence-based mouthguard sensor for volatile sulfur compound (VSC) detection, enabling precise identification of hidden dental lesions [201]. (**d**) A self-powered, switchable chemosensor capable of gas and humidity detection, fabricated using an intelligent and adaptive hydrogel platform [203].

Point-of-care testing (POCT) represents a diagnostic approach designed for rapid, on-site specimen analysis, enabling near-immediate detection of target analytes [195]. By utilizing compact, portable analytical devices in conjunction with pre-calibrated reagents, POCT significantly reduces turnaround time, thereby accelerating clinical decision-making and facilitating timely medical interventions. Previous studies have confirmed that various volatile substances in respiration [196], urine [197], and blood [198] can serve as biomarkers for the early diagnosis of diseases (Figure 4a) [196]. Therefore, the POCT platform based on gas sensors has shown great potential in early disease diagnosis and health assessment due to its fast, inexpensive, non-invasive, and painless characteristics [199]. However, early-stage pathologies often produce extremely weak biological signals that are challenging to detect with conventional analytical techniques.

Furthermore, relying on a single biomarker as an indicator frequently lacks sufficient diagnostic accuracy and robustness, thereby limiting its reliability for comprehensive disease detection and clinical decision-making. Therefore, the demand for multifunctional sensors to achieve multi-dimensional and simultaneous collection of biological signals is increasing daily. For instance, Zhou et al. developed a wearable medical platform that employs non-overlapping pattern gases and strain sensing, which is used to monitor abnormal physiological signals in Parkinson’s patients [200]. Inspired by synaptic structure, bionic sensing layers composed of ZIF-L@Ti_3_CNTx composites with zeolite imidazole salt framework flower-like particles grown in situ on their nanosheets have shown dual-mode high performance in monitoring breath dimethylamine (DMA) gas markers and body dyskinesic tremor in patients with Parkinson’s disease. This intelligent dual-mode sensor is integrated into a flexible circuit, providing forward-looking research results for diagnosing Parkinson’s disease in real-time telemedicine.

Dental caries and periodontitis frequently arise from food impaction and residual debris within the oral cavity, which foster the growth of anaerobic bacterial communities and subsequently damage periodontal tissues. These anaerobic microorganisms metabolically produce ammonia (NH_3_) and volatile sulfur compounds (VSCs), both of which serve as diagnostic volatile biomarkers of oral pathology [201,202]. Electrochemical sensing platforms and optical analytical techniques have been extensively employed for the detection of such exhaled biomarkers, enabling non-invasive diagnosis of oral diseases. For example, Li and colleagues designed a fluorescent sensing material capable of selectively detecting localized VSC concentrations, thereby providing a visual representation of lesion sites with high spatial precision (Figure 4c) [201]. An additional advancement in wearable bioelectronics is the development of skin-conformal, tissue-mimicking sensors. Ding and co-workers recently introduced a wearable skin-interfaced bioelectronic device featuring an electrochemically self-powered architecture based on a flexible, dual-functional hydrogel integrated into a metal–air battery configuration [203]. The hydrogel exhibits a reversible transition between water-rich and water-deficient states, enabling non-invasive and interference-free detection of both oxygen and humidity. This functionality is achieved because oxygen molecules and water vapor directly participate in the oxygen reduction reaction within the hydrogel matrix, acting as either limiting reactants or catalytic modulators that influence reaction kinetics depending on the hydration state. Remarkably, this device demonstrates unprecedented sensing capabilities, achieving sensitivities of 4170.5%/% for oxygen and 380.2%/% RH for humidity, while maintaining a broad detection range and operational stability under variable environmental conditions. Therefore, this device can be combined with wireless communication technology to conduct real-time and remote monitoring of environmental oxygen, percutaneous oxygen pressure changes, breathing, skin moisture, etc., and has important application prospects in safety, health management, and non-contact human–computer interaction.

### 3.3. Agricultural Products Quality Control

**Figure 5:**
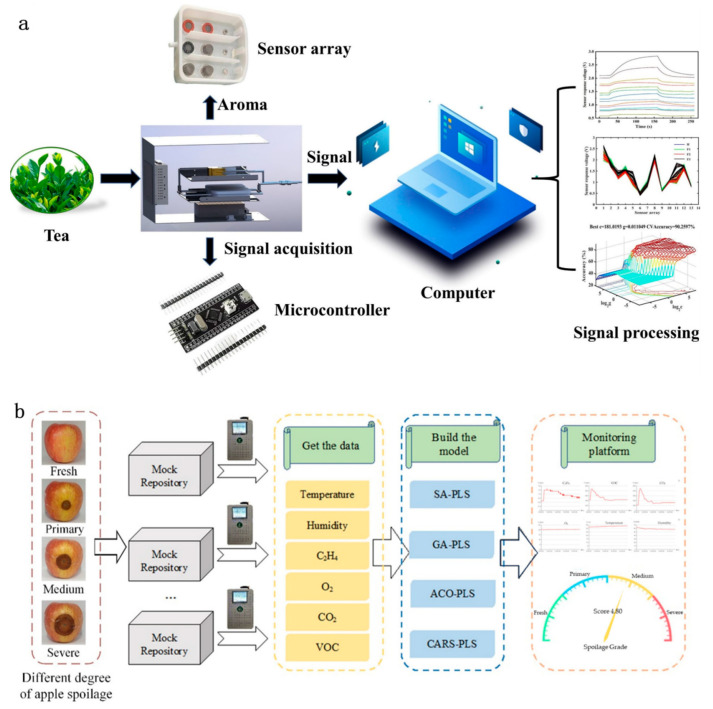

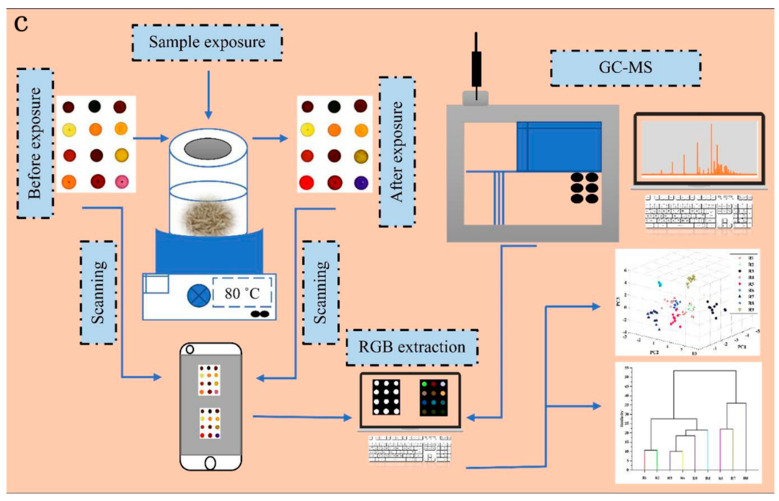
Intelligent gas sensors for the quality control of agricultural products. (**a**) Gas-sensing detection system for real-time monitoring of volatile organic compound variations during oolong tea processing [60]. (**b**) Apple spoilage monitoring and early-warning platform integrating gas sensors with chemometric analysis for storage applications [209]. (**c**) Rice variety discrimination using smartphone-assisted colorimetric sensor arrays combined with gas chromatography-based analytical techniques [47].

The intelligent agricultural quality assessment sensor, as a sensing platform for real-time monitoring of the freshness and quality of agricultural products, has aroused great interest in in situ storage, preparation, and non-in situ supply chains [204,205,206,207]. Han and colleagues developed an advanced gas-sensing platform capable of accurately differentiating between various stages of oolong tea oxidation, thereby improving product quality consistency and enabling greater automation of the processing workflow (Figure 5a) [60]. During oxidation, oolong tea releases a complex mixture of volatile organic compounds (VOCs), including aldehydes, alcohols, and alkenes, which serve as key chemical indicators of process progression. By continuously detecting and analyzing these VOCs, the proposed system facilitates long-term, real-time, online monitoring of the oxidation process, providing valuable insights for process control and quality assurance in tea manufacturing. During the storage and cold chain logistics of agricultural products, due to multiple factors such as physical bumps and microbial infections, it is easy for the microenvironment to deteriorate, leading to large-scale infections and severe post-harvest economic losses. Potential agricultural products, such as fruits and vegetables, when they spoil, emit various volatile components in the surrounding microenvironment. A gaseous biosignature is a telling indicator of storage conditions and is associated with the freshness, quality, and overall consumer acceptability of the produce. Ethylene (C_2_H_4_), isoacetone (CH_3_COCH_3_), methyl fluorene phenol (MBPC; 2,4-dimethyl-1,3-benzyldihydroxybenzene), and a few benzoic acid derivatives, such as pHCA, are the main volatile compounds released during spoilage [208]. These gases are usually colorless, but they have other sensory properties that attribute them to the microbial activity and oxidative processes of food. Yin et al., based on the generation of volatile components during apple spoilage combined with chemometrics analysis, developed a veritable system for monitoring, where early warning is established (Figure 5b) [209]. An integrated gas monitoring array and temperature/humidity sensing system allows for the measurement of volatile organic compounds such as CO_2_, O_2_, and C_2_H_4_. In the end, through multiple modeling methods, Yin et al. established a multi-factor early warning model for apple spoilage, which can realize real-time corruption assessment and provide an important technical guarantee for agricultural product storage management.

Intelligent gas sensor technology also shows excellent potential in the identification of agricultural products [210,211]. Arslan et al. analyzed the aroma components of nine rice varieties through solid-phase microextraction gas chromatography-mass spectrometry (Figure 5c) [47]. They found that there were significant differences in volatile compounds among rice varieties from different geographical sources, and these differences could be used as identification features. Based on this, they developed a colorimetric sensor array system worn on smartphones, which can quickly and cost-effectively identify rice varieties and geographical origins. This system generated a unique color difference image by analyzing the color response characteristics of volatile compounds in rice on the sensor film and combining it with the subsequent mass spectrometry data analysis. Subsequently, they adopted principal component analysis, hierarchical clustering, and a nearest neighbor algorithm to classify and recognize the color difference images. Eventually, they achieved precise identification among different rice varieties, providing an innovative solution for the in situ quality control of agricultural products.

### 3.4. Food Safety Inspection

**Figure 6:**
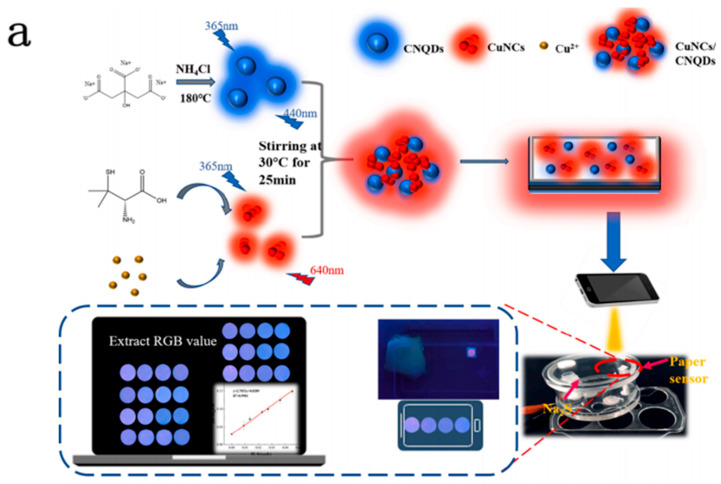

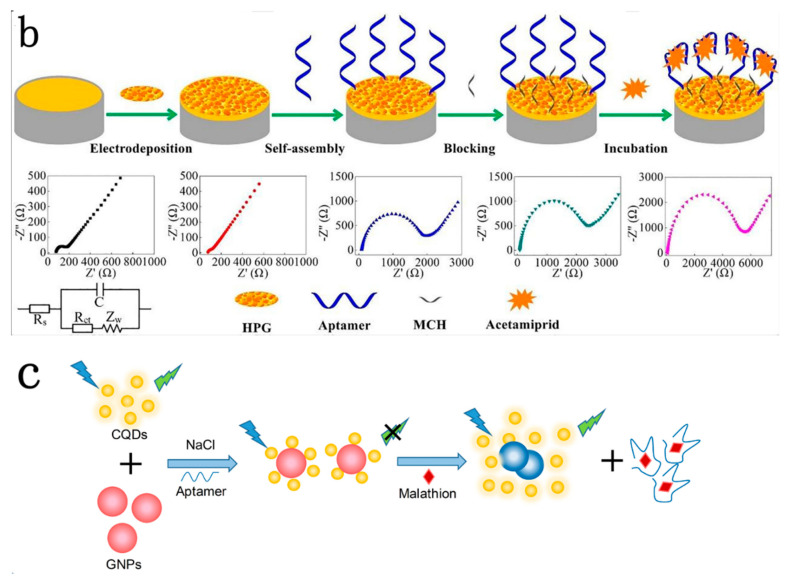
Intelligent gas sensors for food safety detection. (**a**) A fluorescent sensing visual inspection platform for real-time monitoring of meat spoilage [218]. (**b**) Impedimetric aptasensor based on highly porous gold for sensitive detection of acetamiprid in fruits and vegetables [222]. (**c**) Fluorescence and colorimetric dual-mode sensor for visual detection of malathion in cabbage based on carbon quantum dots and gold nanoparticles [223].

The food spoilage sensor in intelligent food safety assessment, a key sensing platform for real-time food freshness and spoilage monitoring, is attracting widespread attention [212,213,214,215,216]. In inspecting meat products, these sensors can provide damage alerts by detecting biomarkers closely related to food decomposition. Among them, total volatile basic nitrogen (TVBN) and its main components—volatile biogenic amines (VBAs), such as putramine, calamine, nitrosamine, n-hexylamine, benzylamine, and -NHET_2_, etc., are protein derivatives produced by the decomposition of meat under the action of microorganisms [217]. These biomarkers can not only reflect the degree of food spoilage but also help inspectors quickly identify microbial contamination in food, ensuring the safety of the products. Huang et al. developed a fluorescence sensing platform for visually detecting hydrogen sulfide (H_2_S) based on proportional fluorescent substances (Figure 6a) [218]. This platform utilizes copper nanoclusters (CuNCs) and nitrogen-doped carbon quantum dots (CNQDs) as dual-emission fluorescent materials, significantly enhancing the detection sensitivity for low-concentration H_2_S. In the concentration range of 0–45.2 ppt of H_2_S, the gas sensor has a very low detection limit of 4.35 ppt, yet with excellent stability and reliability in practical use scenarios. The time-consuming process of detecting H_2_S in spoiled meat has been dramatically reduced, and this work offers a new, highly effective portable strategy for real-time monitoring of hydrogen sulfide release during food preservation. More specifically, intelligent gas sensors are well suited for monitoring potential food and pharmaceutical residues. They heavily depend on the exquisite responses to target contaminants and chemically treated functional materials or nanostructures; therefore, they usually work by detecting specific pollutants through targeted detection [219]. For instance, when detecting key contaminants such as methylamine and cycloformosin, these sensors trigger significant signal changes, including current, optical, and magnetic changes, through their affinity with the target gas, enabling rapid quantitative analysis of trace residues. In addition, this type of sensor, with its outstanding selectivity, stability, and scalability, is widely used in the online detection of key pollutants in food safety supervision, providing a convenient and effective means to ensure the safety of food circulation. Through in-depth optimization of target chemical reactions and signal amplification technology, intelligent gas sensors enhance detection sensitivity and significantly reduce analysis costs, extensively promoting the efficient implementation of food and drug residue monitoring work [220,221]. Xu et al. fabricated a novel electrochemical aptamer sensor modified with high-porosity gold and aptamers, which was used to determine the acetamiprid content in fruits and vegetables (Figure 6b) [222]. This sensor has high selectivity, repeatability, and stability. Under the optimal conditions, it shows a significant linear response characteristic to the acetamiprid concentration range of 0.5 to 300 nmol/L, and its detection limit can reach 0.34 nmol/L. The sensor was applied to determine acetamidine in fruit and vegetable samples. The experimental results showed excellent performance and a good detection effect. Liang et al. established a dual-mode fluorescence/colorimetric sensor based on carbon quantum dots (CQDs) and gold nanoparticles, which can be used to detect malathion in Chinese cabbage visually (Figure 6c) [223]. The fluorescence composite material of this sensor has obvious emission wavelengths of 527 nm and absorption wavelengths of 524 nm. With the increase in malathion concentration, the fluorescence intensity shows an upward trend, while the colorimetric effect shows a downward trend. Through multi-point modeling of fluorescence intensity (R2 = 0.9914) and colorimetric rate (R2 = 0.9608), the fluorescence and colorimetric calibration curves were successfully prepared within the range of 1 × 10^−9^ to 1 × 10^−2^ M. Based on the fluorescence luminance and color changes at different concentrations, standard arrays were constructed, respectively, and combined with the naked eye method, the rapid determination of malathion content in cabbage samples was achieved. The accuracy recovery rate is 89.9–103.4% for the fluorescence method and 88.7–107.6% for the colorimetric method, and the reliability of this method is remarkable. The sensor has good practical application potential, especially in the rapid screening of malathion with a relatively high content in cabbage samples.

### 3.5. Public Safety

In recent years, ecological and public health issues related to household gas leaks, debris from the transportation of dangerous chemicals, industrial accidents, natural disasters, and national security have received increasing attention [224,225]. Large-scale incidents, such as the train derailment involving vinyl chloride in Ohio, recurrent explosions at chemical manufacturing facilities, and volcanic eruptions, underscore the substantial hazards associated with the release of toxic and flammable substances. Such events not only present immediate risks of combustion and secondary explosions but also pose persistent threats to human health due to the dissemination of hazardous chemical residues. Moreover, the deployment of chemical warfare agents in military contexts exemplifies the extreme pathophysiological damage that toxic chemicals can inflict on biological systems, leading to severe and often irreversible health outcomes [226]. In addition, the demand for rapid analysis of these dangerous gases or toxic substances is reflected in public safety and involves multiple aspects, such as environmental protection and resource loss prevention. Therefore, efficient detection technologies for toxic gas leakage, flammable and explosive gases, and nerve agents have significant commercial value and practical application potential [227].

Hydrogen (H_2_), as one of the most promising clean energy alternatives to fossil fuels, plays a crucial role in transportation and utilization. H_2_ sensors, with their excellent performance, such as low ignition energy and wide explosion limit (4% to 75%), have become key equipment to ensure the operation of H_2_ energy systems [228]. Regarding H_2_ sensor materials, palladium is widely regarded as one of the most suitable precious metals for H_2_ sensors due to its high catalytic activity and chemical stability. A typical application is palladium bimetal-modified −In_2_O_3_, palladium nanotube arrays, and palladium nanoparticle-modified graphene. To solve the problem of long response time based on flexible metal-oxide-semiconductor (MOS) sensors, Sun et al. designed a novel PD-modified MOF thin film, MOF-PD, and successfully integrated it into paper circuits, achieving rapid detection of 1–H_2_ [228]. This innovative solution not only enhances the detection sensitivity but also significantly improves the durability and reusability of the sensor. It shows a 155% change in resistance response within 12 s in 10,000 bending cycle tests, providing an efficient and reliable solution for H_2_ leakage monitoring.

### 3.6. Smart Gas Sensor in the IoT

The Internet of Things refers to a networked ecosystem of intelligent devices capable of seamless data exchange through energy-efficient wireless communication protocols [229]. These include short- and long-range technologies such as ZigBee, Bluetooth Low Energy (BLE), LoRa, SigFox, Z-Wave, Wi-Fi, and Near Field Communication (NFC), enabling real-time connectivity and interoperability across diverse applications [230,231,232]. These technologies are widely applied in intelligent gas sensors to achieve real-time collection and transmission of environmental data [233,234]. The IoT-based system can be used to monitor atmospheric air quality remotely. It measures the concentration of pollutants in the air in real time through multiple distributed sensor nodes and transmits the data to the cloud for analysis and visualization processing [235,236,237]. Similarly, in the fight against the epidemic, IoT technology has also been employed to deploy intelligent gas sensor networks, enabling real-time monitoring and early warning of gas hazards in densely populated areas, thereby effectively supporting public health management and emergency response systems [238].

Wireless sensor networks offer substantial improvements in the spatiotemporal resolution of sensing data, thereby facilitating real-time detection even under complex and dynamic environmental conditions [239,240,241]. As an example, Fan and colleagues introduced a self-powered, integrated nanostructured gas sensing platform (SINGOR) utilizing three-dimensional Pd/SnO_2_ films, which was further deployed within a wireless SINGOR network to enable efficient distributed gas monitoring [9]. This sensor has been successfully applied to smart home air quality monitoring and leakage locations. By deploying SINGOR sensor arrays in multiple residence locations and combining the cross-response technology of PCA and SVM algorithms, gases such as H_2_, formaldehyde, toluene, and acetone within the relative humidity range can be accurately identified. Furthermore, Jin et al. proposed the telecommunications technology based on PL-enhanced Fidelity (Li-Fi) in the field of NO_2_ sensors, which improved the sensing performance and achieved low-power remote monitoring and tracking of air pollutants [18]. These construction concepts and applications provide the intelligent environmental monitoring system with more efficient data collection and analysis capabilities.

Integrating wearable bioelectronic gas sensors with IoT frameworks presents significant potential for mitigating the spread of respiratory infectious diseases. For example, Wang and co-workers developed an intelligent wearable mask capable of continuously monitoring airborne viral proteins in real time [242]. The collected data are wirelessly transmitted to a cloud-based IoT platform for advanced analytics, enabling early warning and timely intervention in potential outbreak scenarios. On this basis, the application of AIoT technology enables wearable H_2_ gas sensors to maintain good performance despite the challenges of working in extreme environments, with the help of vision-assisted algorithms and statistical model tools, providing strong support for biosecurity monitoring in bright environments [243,244,245]. These construction concepts provide solutions for health monitoring in daily life and lay a technical foundation for preventing and controlling public health events and the early warning of epidemics.

## 4. Summary and Prospects

Intelligent gas sensor technology has developed remarkably over the past few decades, gradually transforming from the initial rigid portable devices to flexible and wearable intelligent electronic devices. The IoT, driven by advanced algorithms, has strongly driven this evolution, and wireless communication technologies have continuously expanded their application scope. Electrical and photoelectric gas sensors have achieved breakthroughs in their fundamental working principles. Combined with ML algorithms, they can significantly enhance the ability to recognize various gases. In addition, the intelligent gas sensor’s structural design is further optimized, providing a reliable guarantee of its deployment in various application scenarios. The technical innovations include a wide range of applications, such as medical environmental monitoring, industrial safety evaluation, personal health management, and public health emergency response. This has drastically improved the utility, as well as the performance of gas sensing technologies. Despite the challenges of more difficult data processing and restricted device flexibility, they are expected to be solved with technological advancements and continued convergence. These enhancements will continue to expand the use cases and bring new solutions across a wide range of industries.

### 4.1. Sensing Accuracy and Detection Discrimination

For actual gas composition monitoring, the relatively high precision levels of electronic and photoelectric sensors are required to yield dependable results. Unfortunately, environmental fluctuations in temperature and humidity can often cause sensor drift over time in operations, resulting in disparate electronic measurements and reducing repeatability. This drift originates not only due to the natural aging and deterioration of sensor hardware but may be enhanced by errors in colorimetric signal interpretation, leading to a significant effect on the accuracy and reliability of sensing data. To address these technical challenges, researchers began to explore more advanced calibration and data analysis methods to reduce measurement errors and enhance the repeatability of sensor data. ML and DL algorithms are significant in this field. They can capture the influence of environmental changes on sensor performance through large-scale sensor data modeling, thereby achieving precise calibration and optimization. In practical applications, although pattern recognition methods are currently mainly adopted for the intelligent recognition of gas components, the highly complex and diverse nature of air mixtures still poses a challenge to detection accuracy. Modeling and analyzing a large amount of sensor data using deep learning algorithms can effectively address the measurement accuracy problem in complex environments and provide important theoretical support and a technical foundation for developing more efficient and reliable intelligent gas composition recognition systems.

### 4.2. Data Integrity and Reproducibility

In practical applications, the reliability and credibility of gas sensors are usually regarded as closely related to the integrity and repeatability of data, which is the traditional standard for evaluating their performance. However, even if DL models are adopted to train and optimize a large amount of data, this ML-based method may still have a relatively high classification error rate. Therefore, in the case of a small dataset scale, the accuracy of the gas sensor itself and the stability of the operator are crucial. In specific applications such as gas composition monitoring, due to the complexity of equipment and environmental conditions and the limited number of sample options, these factors further intensify the challenge of data reliability, affecting the credibility of sensors in practical scenarios.

### 4.3. Low-Frequency Noise Interference

An electronic gas sensor’s detection limit LOD is a key performance indicator, and its value is influenced by multiple factors, among which the most prominent one is the low-frequency noise generated by the sensor when transmitting signals. Specifically, this kind of noise includes 1/f noise, a phenomenon of vibration weakening over time, and random telegraph noise, which gradually reduces the signal quality while transmitting sensing data to the computing unit, thereby reducing the sensitivity. In large-scale integrated systems composed of multiple and complex sensor arrays, a high signal-to-noise ratio means a stronger anti-interference ability and is an important condition for ensuring the reliability of the sensor system. However, due to the lack of in-depth understanding of the generation mechanism and sources of these low-frequency noises in current research, this issue remains a key difficulty affecting the performance improvement of electronic gas sensors and urgently requires further exploration and resolution.

### 4.4. Inherent Defects and Improvements

Electron-type gas sensors and photoelectric gas sensors each face inherent defects that significantly limit their performance and reliability in practical applications. Electronic-type gas sensors, however, are predominantly deficient in their reliance on an external power source, rendering them ineffective during power interruptions or when the power supply is disrupted, thereby reducing their autonomous functionality. Additionally, their chemically sensitive components are easily susceptible to contamination, aging, and other environmental factors, leading to reduced sensitivity and shortened service life. On the other hand, photoelectric-type gas sensors are predominantly limited by their high dependency on light intensity, with their detection capabilities significantly impaired in low-light or no-light environments. Furthermore, the use of external light sources increases both the cost and maintenance burden of these devices, adding to their economic strain. Future research directions should focus on addressing these issues, such as developing low-power self-sufficient energy supply systems to reduce reliance on external power sources, exploring more stable light source technologies capable of operating in complex environments, and enhancing the anti-contamination and anti-aging properties of sensitive materials. In parallel with these efforts, innovative approaches, including research into nanoscale bipolar structures, development of backside illumination mechanisms, or flexible self-regulating systems, could significantly improve sensor detection sensitivity, stability, and reliability.

### 4.5. Material Science and Digital Integration

The development trend of intelligent electronics and photoelectric gas sensors is at the forefront of the intersection of advanced materials science and digital technology. The new generation of sensors, with the application of innovative materials, has significantly enhanced its sensitivity and selectivity, laying a solid foundation for future applications. Integrating data processing technologies driven by the Internet of Things and artificial intelligence changes how sensor data is collected, analyzed, and utilized, making large-scale and high-efficiency data processing possible. Electronic gas sensors, advanced neural network algorithms such as the K-nearest neighbor algorithm, and convolutional neural network signal calibration and drift compensation significantly enhance the short-term and long-term sensing performance. Photoelectric gas sensors, combining image processing algorithms, threshold detection, and edge detection techniques, enable more accurate colorimetric data extraction and analysis, thereby significantly enhancing the reliability and stability of the sensors. In addition, by rationally choosing an appropriate color model and precisely collecting and deeply analyzing complex color changes, the accuracy of substance concentration measurement has been enhanced, and the detection capability of gas sensors has also significantly improved. The further development of artificial intelligence technology has made data interpretation and anomaly detection more efficient. The Multi-Layer Perceptrons (MLPs) and Support Vector Machines (SVMs) have demonstrated remarkable effectiveness in gas monitoring and environmental early warning systems. The integration of advanced deep learning algorithms, such as convolutional neural networks (CNNs), with photoelectric sensor technologies enables the recognition of complex data patterns. It facilitates real-time data analysis, thereby improving both the reliability and operational efficiency of gas sensing systems. This review highlights the current advancements and prospects of intelligent gas sensing technologies. Rapid progress in materials science, embedded computing, wireless sensor networks, and the IoT has driven the evolution of gas sensors from single-function devices to multifunctional, integrated systems. These innovations have garnered significant attention due to their promising applications in environmental monitoring, medical diagnostics, and industrial process control.

This article provides a comprehensive overview of the development trajectory, recent technological breakthroughs, and future potential of intelligent gas sensing platforms. Recent progress in advanced materials engineering, embedded computing, wireless communication technologies, and the IoT has driven the evolution of gas sensors from simple, single-function devices to highly sophisticated multifunctional platforms. This technological convergence has dramatically expanded their practical utility, facilitating impactful applications in environmental monitoring, clinical diagnostics, and industrial process automation, and has consequently attracted growing interest from both academic and industrial sectors. This review provides a comprehensive assessment of recent advancements in intelligent gas sensing enabled by the convergence of multiple interdisciplinary technologies. It emphasizes notable progress in analytical sensitivity, molecular selectivity, and real-time operational performance while addressing persistent technical challenges and exploring potential avenues for future innovation. By consolidating current research achievements and highlighting critical developmental trends, this work offers a strong theoretical framework and practical insights to inform the design and development of next-generation intelligent gas sensing systems.

## Figures and Tables

**Table 1:** Comparative features of different transducer categories used in electronic gas sensing systems.

Transduction Type	Advantages	Disadvantages
Chemiresistor	Simple configuration and working principle.	It is susceptible to environmental disturbances and is limited by a single output type (such as resistance or current), high operating temperature, cross-sensitivity, aging, and drift.
FET	It has multiple types of output signals, such as drain-source current, threshold voltage, and sub-threshold swing.	It is susceptible to environmental disturbances, cross-sensitive to gases with highly similar structures and properties, and has a poor recovery rate and long-term stability.
Capacitor	Additional measurement capabilities over chemical resistors allow for better selectivity and reliability.	It is easily affected by environmental cleanliness, edge effects, parasitic capacitance, etc.
Inductor	It can be magnetically coupled with an external coil for wireless detection.	The circuit configuration is relatively complex.
